# Gender and AB0 Blood Type Differences in a Unicentric Group of University Professors in Southern Italy Who Received the Vaxzevria COVID-19 Vaccine: A Cross-Sectional Survey of Vaccine Side Effects, Attitudes, and Hesitation

**DOI:** 10.3390/vaccines10030373

**Published:** 2022-02-27

**Authors:** Silvana Mirella Aliberti, Luigi Schiavo, Giovanni Boccia, Emanuela Santoro, Gianluigi Franci, Alessandro Ruggiero, Francesco De Caro, Mario Capunzo

**Affiliations:** 1 Department of Medicine, Surgery and Dentistry “Scuola Medica Salernitana”, University of Salerno, 84081 Salerno, Italy; lschiavo@unisa.it (L.S.); gboccia@unisa.it (G.B.); esantoro@unisa.it (E.S.); gfranci@unisa.it (G.F.); fdecaro@unisa.it (F.D.C.); mcapunzo@unisa.it (M.C.); 2 Dai Dipartimento di Igiene Sanitaria e Medicina Valutativa U.O.C. Patologia Clinica E Microbiologica, Azienda Ospedaliero-Universitaria S. Giovanni di Dio e Ruggi D’Aragona Scuola Medica Salernitana, Largo Cittaà di Ippocrate, 84131 Salerno, Italy; 3 Department of Industrial Engineering, University of Salerno, 84084 Salerno, Italy; ruggiero@unisa.it

**Keywords:** Vaxzevria vaccine, university professors, side effects, attitudes, hesitancy, AB0 blood type, gender difference

## Abstract

Vaccination has been a key protective behavior for COVID-19. This study investigated the clinical status of university professors administered the Vaxzevria COVID-19 vaccine, to monitor for any adverse reaction, and to understand attitude and hesitancy to vaccination. Data were collected through an online survey. The study received approval from the relevant ethics committee “Comitato Etico Campania Sud”. Multivariate logistic regressions were used to calculate significant predictors of the outcomes of interest. A gender and AB0 blood type difference in adverse vaccine reactions was found. The multivariate logistic regression model showed that female gender, city residence, blood type A+ and B−, and chronic underlying medical conditions or comorbidities were more strongly implicated in the occurrence of adverse reactions, whereas blood type 0 Rh+ or blood type A Rh− were protective factors of adverse reactions to the Vaxzevria vaccine. Both genders did not show serious adverse reactions to the Vaxzevria vaccine. Based on our results, we are able to support the hypothesis that AB0 blood type and gender difference appear as predictors of Vaxzevria COVID-19 vaccine reactogenicity. Furthermore, in the study population, the degree of concern and hesitation to undergo vaccination was minimal.

## 1. Introduction

The world has been overwhelmed by one of the most widespread and significant public health crises of recent decades due to a new coronavirus disease 2019 (COVID-19) causing severe acute respiratory syndrome (SARS-CoV-2). On 11 March 2020, the World Health Organization [1] declared that COVID-19 can be defined as a pandemic. The global spread has been rapid and with every infection come new opportunities for the virus to mutate.

Now, two years into the pandemic, the coronavirus is responsible for more than five and a half million confirmed deaths worldwide (as of 15 February 2022), according to John Hopkins University data [2]. Italy, the second nation after Wuhan, China to be hit hard at the start of the pandemic, as of 15 February 2022 reported 12.1 million confirmed cases and 151,000 deaths nationwide [3]. Fundamental to pandemic management are protective behaviors [4] such as social distancing, wearing masks [5,6], antiviral drugs [7], and the COVID-19 vaccine. Vaccination has been a key protective behavior for COVID-19 [8], and played an important role in increasing population immunity, preventing serious disease, and reducing the health crisis [9]. In fact, the rate of confirmed deaths appears to have slowed since the world reached four million vaccines in early July [10]. According to national surveillance data from the first 4 months of the vaccination campaign in Israel, two doses of the COVID-19 vaccine reduced both symptomatic and asymptomatic infections, hospitalizations, serious illness and deaths [11,12]. The efficacy of vaccines has also been highlighted by other studies [9,13,14,15]. Cases of serious adverse reactions after vaccination for COVID-19 have also been reported. In particular, several studies have shown cases of adverse reactions such as thrombosis in connection with thrombocytopenia after COVID-19 vaccination with Vaxzevria (Oxford/AstraZeneca—AZD1222) [16,17,18,19,20]. In Italy on March 15, 2021, the Italian Medicines Agency (AIFA) as a precautionary and temporary measure, banned the use of the Vaxzevria (Oxford/AstraZeneca—AZD1222) COVID-19 vaccine nationwide, due to safety concerns regarding the risk of thrombosis in vaccinated subjects [21]. At the same time, other countries had also suspended the use of this vaccine, pending evaluation by the European Medicines Agency (EMA). Vaccination with Vaxzevria (Oxford/AstraZeneca) was resumed on 19 March 2022, after the EMA’s safety committee evaluated the potential risks and benefits of the vaccine [22]. In Italy, a circular letter from the Ministry of Health dated 11 June 2021, established that the Vaxzevria vaccine should be administered only to persons aged 60 years or older and booster shots for those under 60 years should be given with a mRNA vaccine [23].

Given these premises, the objectives of the study were to delineate the clinical status of the southern Italian university population undergoing COVID-19 vaccination with Vaxzevria, to monitor possible adverse reactions to the vaccine, and to understand the attitude and hesitancy to vaccination of the sample examined.

## 2. Materials and Methods

### 2.1. Study Design and Sampling

The study is a cross-sectional survey conducted in the 2021 academic year, from May to August, on a random sample of professors at the University of Salerno, Italy, who took Vaxzevria (Oxford/AstraZeneca) vaccine [24]. The survey population consisted of professors who received the first dose of Vaxzevria on 3 March 2021.

**Figure 1:**
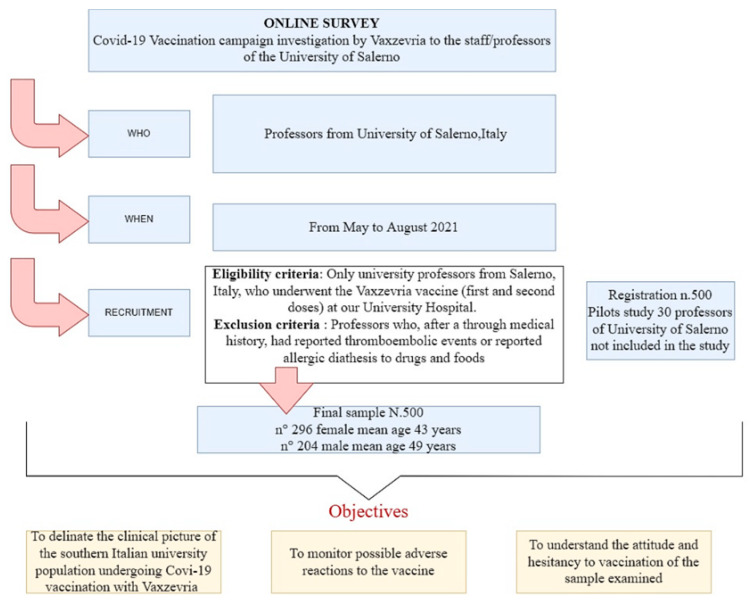
Survey design, including data collection, participant recruitment, and objectives. Note: For more information look at the introduction, study design and results.

Eligibility criteria included only university professors from Salerno, Italy, who underwent the Vaxzevria vaccine (first and second doses) at our University Hospital. Exclusion criteria included professors who, after a thorough medical history, had reported thromboembolic events or reported allergic diathesis to drugs and foods (Figure 1).

The sample size was calculated with the following equation [25]:

(1) $$n=\frac{Z^{2}\;P\left(1-\;P\right)}{d^{2}}$$

where *n* is the sample size, *Z* is *Z* statistic related confidence level, *P* the expected prevalence or proportion, and *d* the precision. In our study the *Z* value is 1.96 for a 95% confidence level, the prevalence is 20% (in proportion of one *P* is equal to 0.2) of professors who could have had serious thrombotic effect, the level of precision is 4% (in proportion of one *d* = 0.04) and the sample size recommended was 384.

### 2.2. Data Collection Procedure

Research participants were invited to participate in the survey through the departmental webpage of Medicine, Surgery, and Dentistry and through the official Facebook social page of the University of Salerno. The survey response rate cannot be calculated because according to University of Salerno protocol, it is not possible to send emails with the “all users” formula, used by the University for service communications to all staff. As mentioned earlier, the only communication channels, for the University’s internal online surveys, are the University’s official Facebook social pages and the Medicine, Surgery and Dentistry departmental website.

Data were collected via a professional online survey platform (LimeSurvey project, Hamburg, Germany), which provides: (1) an intuitive interface for data entry; (2) audit trails to monitor data manipulation and export procedures; (3) automated export procedures for downloading data into common statistical packages; and (4) procedures for importing data from external sources.

The online survey was anonymous and self-reported; the only socio-demographic items required were gender, age, and living location.

An implicit statement of consent was obtained from the participants, as the questionnaire was administered through an electronic tool, on which the professors had to specifically and intentionally access on the Internet through a link (https://covid-19-vaccination.limequery.com, accessed on 15 September 2021). However, in the header of the questionnaire web page, to exclude any liability, the text explained the objective of the study and the anonymous and voluntary nature of participation.

### 2.3. Ethical Approval

The study was designed and performed in accordance with the Declaration of Helsinki. The protocol included full assurance of anonymity, discretion of participation, and absence of risk, conflict of interest, and incentives for participants. The study received approval from the relevant ethics committee “Comitato Etico Campania Sud”, protocol no. 0098507 of 17 May 2021.

### 2.4. Data Collection Instrument

The questionnaire was constructed by the research team, following two focus groups to outline its content. To ensure reliability and validity, the questionnaire was pretested with a random sample of 30 professors from the University of Salerno (Figure 1). After the pre-test, a few modifications were made to further improve the comprehensibility of the questionnaire. The results of the pretest were not included in the study. After the pilot test the entire research team approved the final version of the questionnaire.

The instrument consisted of three main sections: (1) socio-demographic characteristics of the respondent (gender, age, live in a city or suburban area); (2) health or disease status of vaccinees (perception of current health status, presence or absence of chronic underlying medical conditions, comorbidities or disabilities, AB0 blood type, perceived health status after first dose of vaccine, perceived health status after second dose of vaccine). Questions included “yes”, “no”, multiple-choice responses and a horizontal analog scale (rating scales) that took values between 0 (worst condition) and 100 (best condition); (3) vaccine prophylaxis and possible reactions (already had other vaccines yes/no, which pharmaceutical company vaccine was administered, what adverse reactions after vaccine inoculation, no attitude, and hesitation to vaccine). Participants were able to indicate more than 1 response regarding adverse reaction after vaccine inoculation. Questions included “yes” or “no”, open ended question, multiple responses and five-point Likert scales were used, with the end-points labeled as 1 = strongly disagree and 5 = strongly agree.

### 2.5. Statistical Analysis

Descriptive statistics were used to summarize participant characteristics, and responses to all items were shown with absolute and relative frequencies for categorical variables and mean and standard deviation for continuous variables. Univariate analyses were performed using chi-square association tests for categorical variables and t tests for continuous variables to delineate relationships between the outcomes of interest and various characteristics. Next, variables with a *p*-value of 0.25 were entered into the multivariate regression models, and the significant level choices for inclusion and elimination of variables in the models were *p*-values of 0.2 and 0.4, respectively, in agreement with Hosmer and Lemeshow [26]. Multivariate logistic regressions was used to calculate significant predictors of the following four outcomes: Adverse vaccine reactions, ranging from 0 reactions to 12 reactions combined, and which was dichotomized into very common/common reactions = 0 (pain and swelling at the injection site, redness at the injection site, fatigue, headache, muscle and joint pain, chills, nausea, feeling unwell) and uncommon reactions = 1 (drowsiness, dizziness, fever, difficulty falling asleep), in agreement with the classification of adverse reactions to AstraZeneca vaccine established by the European Medicines Agency [27] (Model 1); gender difference in adverse vaccine reactions, measured in male = 0 and female = 1 (Model 2); concern that the COVID-19 vaccine may not be safe, which was dichotomized into no concern (not at all = 0) versus concern (little, so-so, quite a bit, very much = 1) (Model 3); hesitation regarding administration of the COVID-19 Vaxzevria vaccine which was dichotomized as 1 if the answer was “Yes” and 0 if it was “No” (Model 4). The following selected independent variables were included in the logistic regression models: gender (male = 0; female = 1); age, in years (continuous); live (city = 1; suburban area = 0); perception of current health status (continuous); chronic underlying medical conditions, comorbidities (No = 0; Yes = 1); disabilities (No = 0; Yes = 1); blood types were measured 1 type 0 Rh− (baseline result), 2 type 0 Rh+, 3 type A Rh−, 4 type A Rh+, 5 type B Rh−, 6 type B Rh+, 7 type AB Rh−, and 8 type AB Rh+; perceived health status after first dose of vaccine (continuous); perceived health status after second dose of vaccine (continuous); adverse reactions after vaccine inoculation (all responses 1 through 12 were dichotomized into No = 0, Yes = 1; questions related to itching at the injection site, taste disturbance, lymph node enlargement, muscle weakness on one side of the face (acute peripheral facial palsy), and anaphylactic shock were not considered because there were zero responses); concern that the COVID-19 vaccine may not prevent disease (not at all = 0; little, so-so, quite a bit, very much = 1); confidence in the information received about the COVID-19 vaccine (No = 1; Yes = 0). Odds ratios (ORs) and their 95% confidence intervals (CI) were used in the multivariate logistic regression models to measure the independent associations between the different variables and the outcomes of interest. For all analyses, 2-sided *p*-values of 0.05 or less were considered statistically significant. Data analyses were conducted using STATA [28].

## 3. Results

### 3.1. Socio-Demographic and Anamnestic Characteristics

The sample consisted of 500 professors from the University of Salerno who agreed to be interviewed. Nearly 60% of the sample were females, with a mean age of 43 years, two-thirds of them lived in the suburban area, the remaining 40% were male, had a mean age of 49 years, and equally divided between city and suburban residents. Responses about perceptions of current health status, chronic underlying medical conditions, comorbidities, and disabilities were very similar between female and male professors. Most of the respondents belonged to blood type 0 Rh± of which 52.2% were male and 37.8% were female. Numerous were also females belonging to blood type A Rh± (27.7%) and B Rh± (34.1%). The characteristics of the respondent population are summarized in Table 1.

### 3.2. Gender Difference in Adverse Reactions to Vaxzevria Vaccine

**Figure 2:**
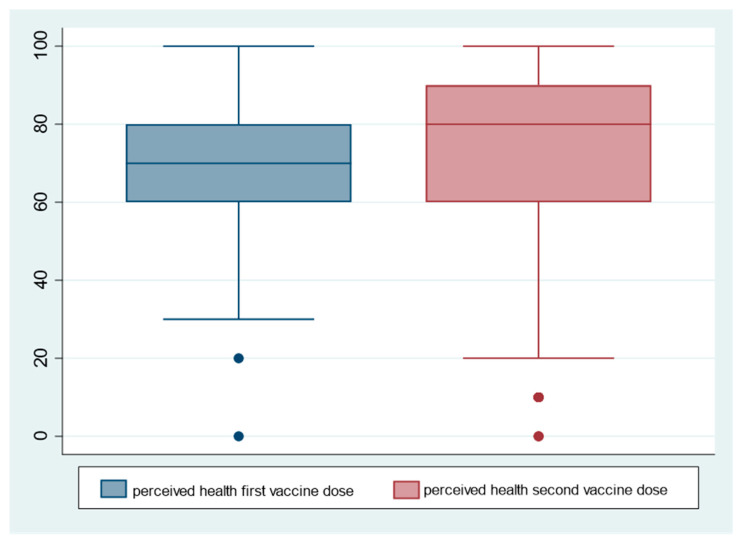
Difference in health perception after the first and second doses of Vaxzevria on the outcome of males and females.

The entire sample examined had received mandatory vaccinations during their lifetime and had never experienced adverse reactions to the vaccinations administered, with the exception of two females and one male of the total sample. After Vaxzevria vaccine inoculation, 55.7% of female professors and 32.5% of male professors experienced adverse reactions. There was a gender difference between males and females in perceived health after the first and second doses of the Vaxzevria vaccine. With both the first and second doses, the health of males (mean 74.4 first dose, 77.1 s dose) was better than that of females (mean 71 first dose, 76.3 s dose), however, both groups perceived better health with the second dose than the first (Figure 2). Table 2 presents the results of the multivariate logistic models predicting the different outcomes of interest. The multivariate logistic regression model, built to study adverse reactions to the vaccine, showed that eight variables were statistically related to outcome. Of these, female gender, city residence, blood type A+ and B−, and chronic underlying medical conditions, comorbidities were most strongly implicated in the occurrence of adverse reactions, whereas blood type 0 Rh+ or blood type A Rh− and disabilities were protective factors of adverse reactions to the Vaxzevria vaccine (Model 1 in Table 2). Both genders did not show severe adverse reactions to the Vaxzevria vaccine, but females showed more reactions of mild and moderate severity than males. The results of the multivariate logistic regression model showed that females had higher occurrence events for injection site pain and swelling, fatigue, chills, dizziness, nausea, and feeling unwell after the Vaxzevria vaccine (Model 2 in Table 2).

### 3.3. Attitude to COVID-19 Vaccine

When assessing attitudes toward the COVID-19 vaccine, 74% of males and 78% of females expressed concern that the vaccine may not be safe. The logistic regression model showed that attitudes of concern were not related to gender, age, or the presence or absence of chronic pathologies or disabilities, but concern was closely related to current health status, perception of one’s health after the first dose of vaccine, and fear that the vaccine may not prevent disease (Model 3 in Table 2). Despite concerns that the COVID-19 vaccine may not be safe and may not prevent disease, only one-third of respondents, 35% male and 31% female, were hesitant toward COVID-19 vaccination. Hesitancy was due to lack of confidence in the information received about the COVID-19 vaccine. The results of the multivariate logistic regression model revealed that respondents’ statistically significant predictors of the hesitation regarding administration of the COVID-19 vaccine included chronic pathologies, disabilities, current health status and lack of confidence in the information received about the COVID-19 vaccine (Model 4 in Table 2).

## 4. Discussion

The main objectives of this study were to investigate the clinical status of university professors, who were subjected to the Vaxzevria COVID-19 vaccine, and to understand whether there was a relationship between adverse reactions to the vaccine and the predictive factors considered. The report on the attitudes of the Italian university population vaccinated against COVID-19 and the regressors of their hesitancy to receive the vaccine was also presented.

Several COVID-19 vaccines are now licensed [29], and the success of a launch often depends on people’s willingness to accept any of them. Vaxzevria (Oxford/AstraZeneca) is a vaccine that aims to prevent COVID-19 infection in the human population [30]. The technology used for the development of the Vaxzevria vaccine uses a virus belonging to the adenovirus family, genetically modified with a gene encoding a specific sars-CoV-2 protein [27]. In terms of unwanted side effects, some studies have shown that most of them are of little clinical relevance. Among the most relevant clinical effects, episodes of facial paralysis, thrombosis in association with thrombocytopenia and Guillain-Barrè syndrome have been reported in 1 in 1000 and 1 in 10,000 people, respectively. This has led the health authorities responsible for pharmacovigilance not to allow the use of the Vaxzevria vaccine in those subjects who have experienced thrombosis with thrombocytopenia syndrome (TTS) or capillary leak syndrome following the administration of Vaxzevria. Furthermore, some cases of allergic reactions, including anaphylaxis, have been reported following the administration of the Vaxzevria vaccine [27].

We highlight that the population included in the present study, consisted of professors who received the first dose of Vaxzevria on 3 March 2021, before the vaccine was suspended by the EMA to assess its potential benefits and risks, and the second dose on 3 June or 11 June 2021 (the sample was divided into two groups for the administration of the second dose), before the circular of the Ministry of Health recommended the use of the Vaxzevria vaccine after the age of 60 years, and despite this we emphasize that the sample examined did not manifest serious side effects but only mild or moderate. Moreover, according to our results, this study indicates that adverse reactions to the Vaxzervria vaccine depend on several variables such as gender, AB0 blood type, and chronic pathologies. Interestingly, we found that female respondents, living in cities, blood type A+ and B−, and chronic pathologies were most implicated in the occurrence of adverse reactions, whereas blood type 0 Rh+ or blood type A Rh− could be protective factors of adverse reactions to Vaxzervria vaccine. It has been anecdotally observed that AB0 blood type may impact the severity of side effects experienced by those receiving mRNA vaccination for COVID-19. However, a retrospective cross-sectional survey of 33,000 frontline healthcare workers, students, and volunteers to determine if there was a relationship between vaccination reactogenicity and AB0 blood type shows no statistically significant association between any blood type and any side effect for either COVID-19 mRNA vaccine [31]. Therefore, the authors conclude that COVID-19 mRNA vaccination may cause significant reactogenicity, but ABO blood type does not appear to be a predictor of vaccine reactogenicity. In addition, here we found that both females and males did not show severe adverse reactions to the Vaxzevria vaccine, but females compared to males showed more reactions of mild and moderate severity. Specifically, we found that females had a statistically significant probability of risk for injection site pain and swelling, fatigue, chills, dizziness, nausea, and feeling unwell after Vaxzevria vaccine. These findings are in agreement with previous works reporting lower adverse effects in males [32].

Vaccine hesitancy is a major barrier in achieving herd immunity in different populations. Regarding attitudes toward the COVID-19 vaccine, in the present study we found that attitudes of concern were not related to gender, age, or the presence or absence of chronic pathologies or disabilities, but concern was closely related to current health status, perception of one’s health after the first dose of vaccine, and fear that the vaccine may not prevent disease. Despite these concerns only one-third of respondents were hesitant toward COVID-19 vaccination, thus they showed a greater willingness to be vaccinated. Hesitation was primarily due to lack of confidence in the information received about the COVID-19 vaccine and perception of current health status. Furthermore, it is noteworthy to point out that participants were vaccinated at a time when the COVID-19 vaccine was strongly recommended but not mandatory. Compulsory vaccination has only recently been introduced in Italy for public administration workers and those over 50 years of age. These findings are in agreement with previous work that reported that through a variety of different factors contributed to increased hesitancy, having negative perception of vaccine efficacy, safety, and convenience, are the most frequent [33,34].

Regarding the limitations of the study, we can say that the results of this investigation must be interpreted with the following potential methodological limitations. First, the cross-sectional design used may limit the ability to identify causal relationships between the different independent variables and the different outcomes of interest. Second, the generalizability of the results may be limited. The selected population included only the team of university professors from Southern Italy, and this may not reflect the attitudes and reactogenicity to the vaccine of the general population of the country as a whole. In addition, we asked participants during the interview if they had any underlying chronic medical conditions or comorbidities, the specification of which was not investigated.

## 5. Conclusions

Based on our results, we can support the hypothesis that gender difference and AB0 blood type appear to be predictors of Vaxzevria COVID-19 vaccine reactogenicity. Obviously, the present study is not large enough to provide statistically valid conclusions, and further and larger studies are necessary to confirm our preliminary data. Furthermore, in the study population, the degree of concern and hesitation to undergo vaccination was minimal. Additional larger, multicenter, randomized clinical trials are needed to confirm these preliminary data.

## Figures and Tables

**Table 1:** Respondents’ socio-demographic and anamnestic characteristics.

Characteristics of Respondents (Tot Sample = 500)	Female N	%	Male N	%
Gender	296	59.2	204	40.8
Age in year	43 ± 9.2 (26–66) *		49 ± 7.7 (33–66) *	
Live				
- Suburban area	197	66.5	103	50.7
- City	99	33.5	100	49.3
Perception of current health status (0–100)	87 ± 11.6 (60–100)		86 ± 11.0 (50–100)	
Chronic underlying medical conditions, comorbidities				
- No	228	77.1	154	75.9
- Yes	68	22.9	49	24.1
Disability				
- No	280	94.6	195	96.1
- Yes	16	5.4	8	3.9
Blood type				
- Group 0 Rh−	33	11.1	43	21.2
- Group 0 Rh+	77	26	63	31.0
- Group A Rh−	8	2.7	11	5.5
- Group A Rh+	73	24.7	40	19.7
- Group B Rh−	41	13.9	8	3.9
- Group B Rh+	60	20.3	25	12.3
- Group AB Rh−	1	0.3	0	0
- Group AB Rh+	3	1	13	6.4
Had other vaccines during your lifetime				
- No	0	0	0	0
- Yes	296	100.0	204	100.0
Had adverse reaction to vaccines given during your lifetime				
- No	294	99.3	203	99.5
- Yes	2	0.7	1	0.5
Adverse reactions after inoculation of the Vaxzevria vaccine				
- No	131	44.3	137	67.5
- Yes	165	55.7	66	32.5
Specific adverse reactions after inoculation of the Vaxzevria vaccine				
- Pain and swelling at the injection site	175	59.1	87	42.8
- Redness at the injection site	31	10.4	17	8.3
- Itching at the injection site	0	0	0	0
- Fatigue	191	64.5	105	51.7
- Drowsiness	69	23.3	50	24.6
- Headache	169	57.1	111	54.6
- Muscle and joint pain	110	37.1	60	29.5
- Chills	161	54.4	49	24.1
- Dizziness	4	1.3	7	3.4
- Fever	146	49.3	64	31.5
- Nausea	37	12.5	6	2.9
- Taste disturbances	0	0	0	0
- Swollen lymph nodes	0	0	0	0
- Difficulty falling asleep	10	3.4	5	2.4
- Feeling unwell	102	34.4	53	26.1
- Weakness of the muscles on one side of the face (acute peripheral facial paralysis)	0	0	0	0
- Anaphylactic shock	0	0	0	0

Note: N total observations professor sampling, divided in gender difference; * Mean ± Standard deviation (Range). Number for each item may not add up to total number of study population due to missing values.

**Table 2:** Results of multivariate logistic regression analysis examining outcomes of interest based on several explanatory variables.

Variable	OR	95% CI	*p*
Model 1. Adverse vaccine reactions (Sample size = 498) Log likelihood = −276.4, x^2^ = 134.62 (11 df), *p* < 0.001			
Females	2.82	1.80–4.42	<0.001
Age in years	1.44	0.74–2.82	0.277
City	1.63	1.05–2.52	0.026
Blood type			
- 0 Rh−	1 ^a^		
- 0 Rh+	0.38	0.20–0.73	0.003
- A Rh−	0.20	0.05–0.81	0.025
- A Rh+	2.94	1.51–5.74	0.002
- B Rh−	3.94	1.63–9.56	0.002
- B Rh+	0.52	0.26–1.05	0.072
- AB Rh−	---	---	---
- AB Rh+	1.81	0.56–5.82	0.318
Chronic underlying medical conditions, comorbidities	2.64	1.50–4.64	0.001
Disabilities	0.23	0.07–0.68	0.008
Model 2. Gender difference in adverse vaccine reactions (Sample size = 500) Log likelihood = −279.8, x^2^ = 116.50 (12 df), *p* < 0.001			
Adverse reactions after inoculation of the Vaxzevria vaccine			
- Pain and swelling at the injection site	2.01	1.31–3.09	0.001
- Redness at the injection site	1.34	0.65–2.74	0.415
- Fatigue	2.27	1.45–3.56	<0.001
- Drowsiness	1.29	0.75–2.22	0.349
- Headache	0.76	0.48–1.20	0.247
- Muscle and joint pain	1.11	0.65–1.89	0.684
- Chills	5.56	3.42–9.03	<0.001
- Dizziness	0.19	0.04–0.87	0.032
- Fever	1.35	0.85–2.16	0.197
- Nausea	11.38	4.06–31.89	<0.001
- Difficulty falling asleep	0.82	0.18–3.71	0.800
- Feeling unwell	1.67	1.04–2.70	0.033
Model 3. Concern that the COVID-19 vaccine may not be safe (Sample size = 500) Log likelihood = −207.6, x^2^ = 131.05 (8 df), *p* < 0.001			
Males	1.24	0.74–2.10	0.404
Age in years	1.94	0.91–4.12	0.084
Chronic underlying medical conditions, comorbidities	0.89	0.43–1.86	0.768
Disabilities	3.52	0.73–16.98	0.116
Perception of current health status	0.94	0.91–0.97	0.001
Perceived health status after first dose of vaccine	0.96	0.94–0.98	0.002
Perceived health status after second dose of vaccine	1.01	0.99–1.02	0.117
Concern that the COVID-19 vaccine may not prevent disease	2.68	2.11–3.42	<0.001
Model 4. Hesitation regarding administration of the COVID-19 Vaxzevria vaccine (Sample size = 500) Log likelihood = −245.8, x^2^ = 140.98 (6 df), *p* < 0.001			
Females	0.72	0.45–1.14	0.163
Age in years	0.84	0.42–1.68	0.633
Chronic underlying medical conditions, comorbidities	2.74	1.44–5.24	0.002
Disabilities	16.17	4.44–58.84	<0.001
Perception of current health status	1.07	1.04–1.10	<0.001
Lack of confidence in the information received about the COVID-19 vaccine	7.43	4.65–11.86	<0.001

Note: 1 ^a^ reference category.

## Data Availability

Not applicable.

## References

[1] World Health Organization (WHO) Director-General’s Opening Remarks at the Media Briefing on COVID-19—11 March 2020. https://www.who.int/dg/speeches/detail/who-director-general-s-opening-remarks-at-the-media-briefing-on-covid-19---11-march-2020.

[2] COVID-19 Dashboard John Hopkins University of Medicine Coronavirus Resource Center on 15 February 2022. https://coronavirus.jhu.edu/map.html.

[3] Our World in Data Statistics and Research. Coronavirus Pandemic (COVID-19)—The Data. https://ourworldindata.org/coronavirus-data.

[4] Bish A., Michie S. (2010). Demographic and attitudinal determinants of protective behaviours during a pandemic: A review. Br. J. Health Psychol..

[5] Aliberti S.M., De Caro F., Boccia G., Capunzo M. (2020). Ist die Corona-Krise eine Lehrmeisterin für die Zukunft? Italienische Erfahrungen im Rahmen weltweiter Diskurse. Z. Evidenz Fortbild. Qual. Gesundh..

[6] Aliberti S.M., De Caro F., Boccia G., Caruso R., Capunzo M. (2021). Dealing with COVID-19: Lessons Learned from the Italian Experience. Coronaviruses.

[7] Sharma O., Sultan A.A., Ding H., Triggle C.R. (2020). A Review of the Progress and Challenges of Developing a Vaccine for COVID-19. Front. Immunol..

[8] WHO (2021). COVID-19 Strategic Preparedness and Response Plan: Operational Planning Guideline: 1 February 2021 to 31 January 2022.

[9] Voysey M., Clemens S., Madhi S.A., Weckx L.Y., Folegatti P.M., Aley P.K., Angus B., Baillie V.L., Barnabas S.L., Bhorat Q.E. (2021). Oxford COVID Vaccine Trial Group. Safety and efficacy of the ChAdOx1 nCoV-19 vaccine (AZD1222) against SARS-CoV-2: An interim analysis of four randomised controlled trials in Brazil, South Africa, and the UK. Lancet.

[10] Istituto Superiore di Sanità Epicentro -Epidemia COVID-19. Aggiornamento Nazionale 21 luglio 2021. https://www.epicentro.iss.it/coronavirus/bollettino/Bollettino-sorveglianza-integrata-COVID-19_21-luglio-2021.pdf.

[11] Antonelli M., Penfold R.S., Merino J., Sudre C., Molteni E., Berry S., Canas L.S., Graham M.S., Klaser K., Modat M. (2022). Risk factors and disease profile of post-vaccination SARS-CoV-2 infection in UK users of the COVID Symptom Study app: A prospective, community-based, nested, case-control study. Lancet.

[12] Menni C., Klaser K., May A., Polidori L., Capdevila J., Louca C.H., Nguyen L.H., Drew D.A., Merino J., Hu C. (2021). Vaccine side-effects and SARS-CoV-2 infection after vaccination in users of the COVID Symptom Study app in the UK: A prospective observational study. Lancet Infect. Dis..

[13] Knoll M.D., Wonodi C. (2021). Oxford-AstraZeneca COVID-19 vaccine efficacy. Lancet.

[14] Ramasamy M.N., Minassian A.M., Ewer K.J. (2020). Safety and immunogenicity of ChAdOx1 nCoV-19 vaccine administered in a prime-boost regimen in young and old adults (COV002): A single-blind, randomised, controlled, phase 2/3 trial. Lancet.

[15] Pormohammad A., Zarei M., Ghorbani S., Mohammadi M., Razizadeh M.H., Turner D.L., Turner R.J. (2021). Efficacy and Safety of COVID-19 Vaccines: A Systematic Review and Meta-Analysis of Randomized Clinical Trials. Vaccines.

[16] Barral M., Arrive L., El Mouhadi-Barnier S., Cornelis F.H. (2021). Thromboaspiration and fibrinolysis infusion for portomesenteric thrombosis after AstraZeneca COVID-19 vaccine administration. Int. Care Med..

[17] Greinacher A., Thiele T., Warkentin T.E., Weisser K., Kyrle P.A., Eichinger S. (2021). Thrombotic thrombocytopenia after ChAdOx1 nCov-19 vaccination. N. Engl. J. Med..

[18] Taquet M., Husain M., Geddes J.R., Luciano S., Harrison P.J. (2021). Cerebral venous thrombosis and portal vein thrombosis: A retrospective cohort study of 537,913 COVID-19 cases. EClinicalMedicine.

[19] Saleh A., Collins J. (2021). Case study of thrombosis and thrombocytopenia syndrome following administration of the AstraZeneca COVID-19 vaccine. Aust. J. Gen. Pract..

[20] Oldenburg J., Klamroth R., Langer F., Albisetti M., von Auer C., Ay C., Korte W., Scharf R.E., Pötzsch B., Geinacher A. (2021). Diagnosis and Management of Vaccine-Related Thrombosis following AstraZeneca COVID-19 Vaccination: Guidance Statement from the GTH. Hamostaseologie.

[21] AIFA AIFA: Sospensione Precauzionale del Vaccino AstraZeneca, Published 15 March 2021. https://www.aifa.gov.it/-/aifa-sospensione-precauzionale-del-vaccino-astrazeneca.

[22] EMA COVID-19 Vaccine AstraZeneca: Benefits Still Outweigh the Risks Despite Possible Link to Rare Blood Clots with Low Blood Platelets. https://www.ema.europa.eu/en/news/covid-19-vaccine-astrazeneca-benefits-still-outweigh-risks-despite-possible-link-rare-blood-clots.

[23] Ministry of Health Circular Letter dated 11 June 2021. https://www.trovanorme.salute.gov.it/norme/renderNormsanPdf?anno=2021&codLeg=81053&parte=1%20&serie=null.

[24] Epicentro ISS Vaxzevria (ChAdOx1-S), il Vaccino Contro il COVID-19 Sviluppato da Università di Oxford e AstrZeneca. https://www.epicentro.iss.it/vaccini/covid-19-vaccino-astrazeneca.

[25] Pourhoseingholi M.A., Vahedi M., Rahimzadeh M. (2013). Sample size calculation in medical studies. Gastroenterol. Hepatol. Bed Bench..

[26] Hosmer D.W., Lemeshow S. (2000). Applied Logistic Regression.

[27] EMA European Medicines Agency. Vaxzevria, COVID-19 Vaccine (ChAdOxl-S [Recombinant]). https://www.ema.europa.eu/en/documents/product-information/vaxzevria-previously-covid-19-vaccine-astrazeneca-epar-product-information_en.pdf.

[28] StataCorp (2019). Stata Statistical Software: Release 16.1.

[29] Meo S.A., Bukhari I.A., Akram J., Meo A.S., Klonoff D.C. (2021). COVID-19 vaccines: Comparison of biological, pharmacological characteristics and adverse effects of Pfizer/BioNTech and Moderna Vaccines. Eur. Rev. Med. Pharmacol. Sci..

[30] Marfe G., Perna S., Shukla A.K. (2021). Effectiveness of COVID-19 vaccines and their challenges (Review). Exp. Ther. Med..

[31] Allan J.D., McMillan D., Levi M.L. (2021). COVID-19 mRNA Vaccination, ABO Blood Type and the Severity of Self-Reported Reactogenicity in a Large Healthcare System: A Brief Report of a Cross-Sectional Study. Cureus.

[32] Iguacel I., Maldonado A.L., Ruiz-Cabello A.L., Casaus M., Moreno L.A., Martínez-Jarreta B. (2021). Association between COVID-19 Vaccine Side Effects and Body Mass Index in Spain. Vaccines.

[33] Cascini F., Pantovic A., Al-Ajlouni Y., Failla G., Ricciardi W. (2021). Attitudes, acceptance and hesitancy among the general population worldwide to receive the COVID-19 vaccines and their contributing factors: A systematic review. EClinicalMedicine.

[34] Luz P.M., Johnson R.E., Brown H.E. (2017). Workplace availability, risk group and perceived barriers predictive of 2016–17 influenza vaccine uptake in the United States: A cross-sectional study. Vaccine.

